# Chemokines in Innate and Adaptive Granuloma Formation

**DOI:** 10.3389/fimmu.2013.00043

**Published:** 2013-02-25

**Authors:** Stephen W. Chensue

**Affiliations:** ^1^Department of Pathology, University of Michigan Medical SchoolAnn Arbor, MI, USA; ^2^Section of Pathology and Laboratory Medicine, Veterans Affairs Ann Arbor Healthcare SystemAnn Arbor, MI, USA

**Keywords:** granuloma, chemokines, chemokine receptors, innate immunity, inflammation

## Abstract

Granulomas are cellular inflammations that vary widely in histologic appearance depending upon the inciting agent and immunologic status of the responding host. Despite their heterogeneity, granulomas are at their core an ancient innate sequestration response characterized by the accumulation of mononuclear phagocytes. In fact, this innate cellular response was first observed by Metchnikov in simple invertebrates. Among higher vertebrates, environmental pressures have resulted in the evolution of more sophisticated adaptive immune responses which can be superimposed upon and modify the character of granulomatous inflammation. Compared to immune responses that rapidly neutralize and eliminate infectious agents, the granuloma represents a less desirable “fall back” response which still has value to the host but can be co-opted by certain infectious agents and contribute to bystander organ damage. Understanding granulomas requires an analysis of the complex interplay of innate and adaptive molecular signals that govern the focal accumulation and activity of their cellular components. Among these signals, small molecular weight chemoattractant proteins known as chemokines are potentially important contributors as they participate in both directing leukocyte migration and function. This tract will discuss the contribution of chemokines to the development of innate and adaptive granuloma formation, as well as describe their relationship to more recently evolved cytokines generated during adaptive immune responses.

## Introduction

**Figure 1:**
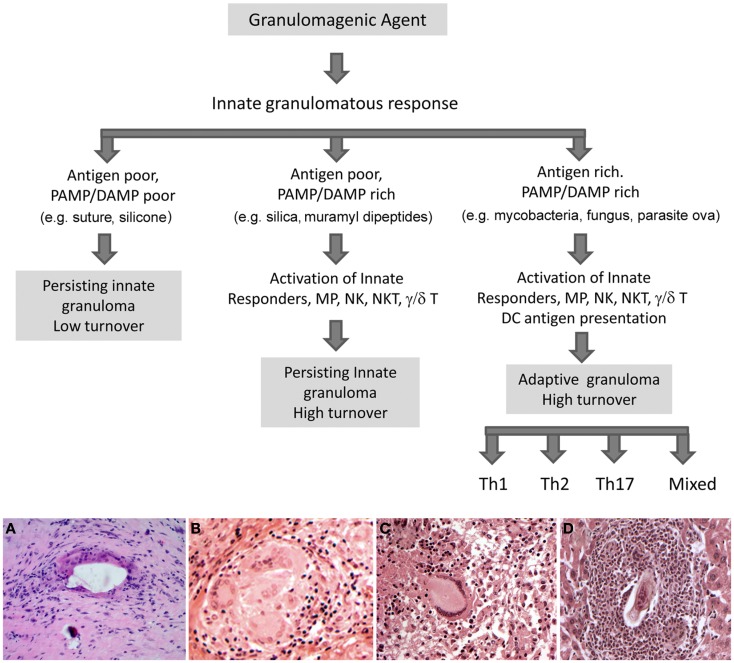
**Potential fates of the innate granuloma**. In the immunocompetent host the ultimate type of granuloma manifested will depend in large part on the nature of the inciting agent. Weakly antigenic agents with poor expression of pathogen (PAMP) and or damage (DAMP) associated molecular patterns will result in a persisting low turnover innate granuloma. Examples would be surgical implant and suture granulomas. Weakly antigenic agents associated with greater PAMPs or DAMPs will activate a broader spectrum of innate responding cells resulting in more complex higher turnover innate granulomas. Examples would be silica, zymosan, and muramyl dipeptide induced granulomas. Strongly antigenic agents with associated PAMP or DAMP signals will initiate additional adaptive antigen-specific T cell responses resulting in a complex high turnover granuloma. The types of cellular components will depend upon the participating T cell subset. Examples would be mycobacterial, fungal, and parasite ova elicited granulomas. It should be noted that humoral elements including antibodies and complement may also influence lesions. Lower panels show examples of the histologic appearance of the various granulomas. **(A)** Innate low turnover foreign-body granulomatous response to surgical bone cement; **(B)** Innate high turnover granulomatous response to silica; **(C)** Adaptive granulomatous response to *M. tuberculosis*, Th1 type; **(D)** Adaptive eosinophil-rich, granulomatous response to schistosome parasite egg, Th2 type. Original magnifications 200×.

The granuloma is defined as a cellular immune response in which the local accumulation of mononuclear phagocytes is a key component (Warren, 1976). The aggregation of mononuclear phagocytes is a sequestration response likely representing one of the earliest forms of innate cellular immunity. It is observed in primitive multicellular organisms and was first documented by Elie Metchnikov in his studies of starfish larvae. Over the millennia more sophisticated innate and adaptive immune responses have evolved, supplanting, or being superimposed upon the granuloma. In modern vertebrates, granulomas have varied histologic appearances and cellular elements depending upon the nature of the eliciting agent and host immune status, which determine the degree of cellular activity and chronicity of the response (Figure 1). In humans, there are many granulomatous conditions indicating that this response has persisted among the spectrum of immune responses and may confer some benefit, but compared to modern adaptive immune responses which can often rapidly eliminate microbes, the granuloma may be a “fall back” response relying on sequestration and attempted elimination. However, some pathogens such as *Mycobacterium tuberculosis* can co-opt the response residing for decades within granulomas only to reemerge when adaptive immunity is compromised. Other undesirable features of granulomas are chronic bystander organ damage and fibrosis. Hence, an understanding of the mechanisms underlying granuloma formation would potentially have important therapeutic benefit.

## Chemokines and Chemokine Receptors

A basic requirement of granuloma formation involves directed cell migration to achieve focal cellular accumulation in response to infection, injury, or foreign bodies. It is well established that cell migration requires surface membrane receptors capable of recognizing extracellular molecules which initiate intracellular signal transduction events leading to selective gene expression and the cytoskeletal reorganization required for cell movement. Directed migration appears to depend upon cells sensing and following molecular chemotactic gradients of increasing concentration generated in the vicinity of an inciting agent. There are many types of chemotactic molecules but a family of homologous proteins with conserved motifs containing four cysteine residues known as chemokines, appear to be of particular importance. The topic of chemokines and their receptors has been extensively reviewed so need only a brief description herein (Zlotnik and Yoshie, 2012). There are over 40 known human chemokine ligands (L) divided among four classes based upon the arrangement of the amino terminal cysteine residues, these are suffixed, CCL, CXCL, CX3CL, and XCL. Most have homologs in the mouse and are produced by a wide variety of host cells. Their carbohydrate free molecular weight ranges from 7 to 10 kDa and they are often basically charged molecules with heparin binding properties. Their activity is mediated by seven-transmembrane domain, G protein-coupled receptors. The primary receptor-binding domain of all chemokines is near the extracellular amino terminus. Over 20 chemokine receptors have been characterized. Some display promiscuous ligand binding while others bind only a single known ligand. Receptor nomenclature is determined by the class of chemokine it binds (CCR, CXCR, CX3CR, and XCR). Receptors are differentially expressed by cell populations and can change during stages of cell maturation/activation. For example, CXCR1 and CXCR2 are highly expressed by neutrophils permitting responses to chemokine ligands CXCL1–8, which express the amino acid sequence of glutamic acid-leucine-arginine (ELR) immediately before the first cysteine of the CXC motif. In contrast, CXCR3 is expressed by effector lymphocytes and responds to CXCL9, 10, and 11, which lack the ELR sequence. Chemokines are expressed in a vast array of pathologic and physiologic states involving cellular migration. Therefore a mechanistic role in granuloma formation is a reasonable hypothesis. This tract will present a focused review of the studies performed in our laboratory to test this hypothesis.

## Experimental Granuloma Models

**Figure 2:**
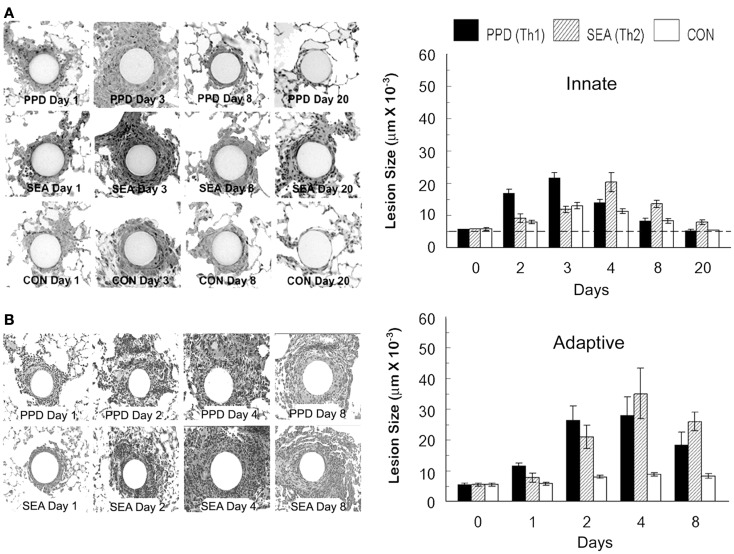
**Synchronized innate and adaptive antigen bead-elicited pulmonary granuloma formation**. **(A)** Histologic appearance and sizes of innate antigen bead granulomas. Agarose beads covalently bound to *Mycobacterium bovis* purified protein derivative (PPD), *Schistosoma mansoni* soluble egg antigens (SEA), or bovine serum albumin (BSA), (CON) were administered intravenously to naïve C57BL/6 mice at a dose of 6000 beads per mouse. Lungs were harvested at the designated time points for histologic examination and cross-sectional measurement using computer assisted image analysis. A minimum of 20 lesions were measured. Dashed line represents average bead size. **(B)** Histologic appearance and sizes of adaptive antigen bead granulomas. Antigen beads were administered as above, but mice were pre-sensitized to the respective mycobacterial and schistosome egg antigens. Lungs were harvested at the designated time points for histologic examination and cross-sectional measurement as above. Size of a non-antigen coated control bead reaction is shown in chart for comparison. Bars are means and standard deviations of five mice.

**Figure 3:**
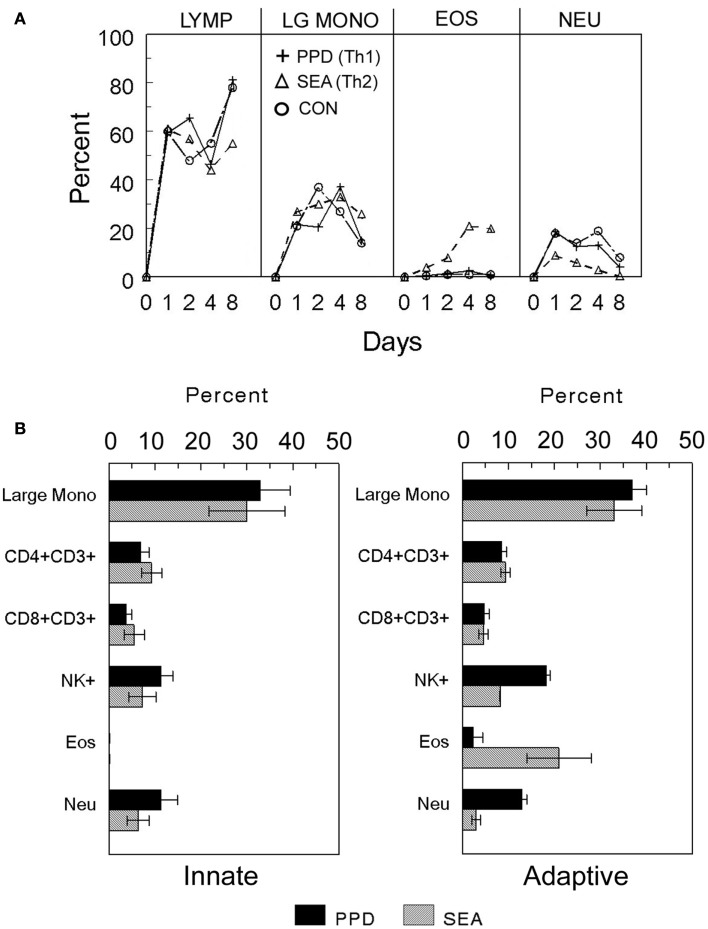
**Cellular composition of innate and adaptive antigen bead-elicited pulmonary granulomas**. **(A)** Time course of major cell populations recruited during adaptive and innate granuloma induction. Innate BSA and adaptive PPD- and SEA-antigen bead lung granulomas were elicited as described in Figure 2. On the indicated days, granulomas were harvested and enzymatically dispersed, washed, and then used for differential analysis. **(B)** Day 4 cell composition of innate and adaptive PPD- and SEA-antigen bead granulomas. Granulomas were elicited and harvested as above and then washed cellular suspensions were subjected to differential analysis to determine cellular components. Lymphocytes (Lymp), large mononuclear cells (Large mono), eosinophils (Eos), and neutrophils (Neu) were determined by standard morphologic analysis. Flow cytometry was used to identify lymphoid subpopulations CD4+ and CD8+ T cells, natural killer (NK+). Bars are means and standard deviations of five mice.

In order to study mechanisms underlying granuloma formation, a number of animal-based experimental approaches have been developed which model a variety of granulomatous conditions, but due to widely varying experimental conditions it is difficult to compare cellular and molecular events among them. To provide a systematic experimental approach, our laboratory developed and characterized mouse models of synchronized lung granuloma formation elicited by intravenous embolization of agarose beads coupled to antigens derived from pathogens associated with granulomatous diseases. Hence, granulomas elicited by diverse etiologic agents could be studied in parallel. As iconic representatives of highly prevalent granulomatous diseases, we utilized purified protein derivative (PPD) from *Mycobacterium bovis* and soluble egg antigens (SEA) derived from ova of the helminth parasite *Schistosoma mansoni*. Detailed immunologic characterization revealed that in pre-sensitized animals, the antigen-bead models respectively elicited polarized Th1 [interferon-γ (IFNγ) mediated] and Th2 (interleukin -4 and -13 mediated) adaptive hypersensitivity-type granulomas with cellular compositions similar to those elicited by live infections (Chensue et al., 1994). As well as being compared to each other, these adaptive responses could be compared to those elicited in unsensitized naive mice, representing the underlying innate granulomatous response. As shown in Figures 2 and 3, innate granulomas are smaller with comparable cellular compositions, whereas the adaptive responses are significantly larger with the PPD response showing greater neutrophil and NK cell component and the SEA response showing greater eosinophil content superimposed upon the large mononuclear infiltrate. The period of most rapid cellular recruitment occurs from 1 to 3 days with a period of sustenance followed by resolution.

## Chemokine Expression Patterns during Innate and Adaptive Granuloma Formation

**Figure 4:**
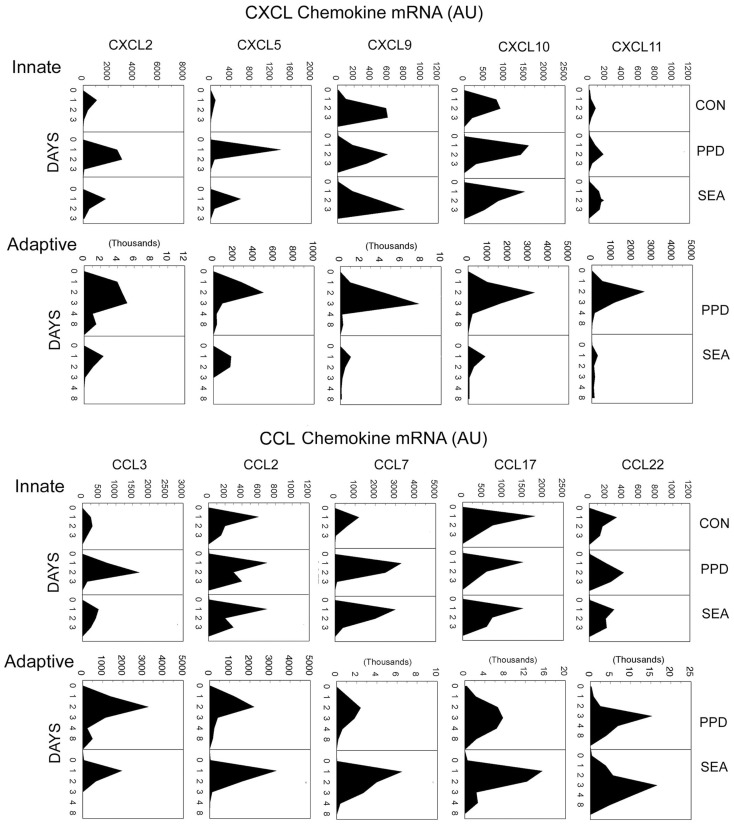
**Dynamics of chemokine transcript expression during innate and adaptive antigen bead-elicited granulomas formation**. Innate and adaptive PPD- and SEA-antigen bead granulomas were elicited as described in Figure 2. Lungs were harvested at the designated time points for mRNA extraction and cDNA preparation. Chemokine transcript levels were determined by semiquantitative real-time RT-PCR. Mean arbitrary units (AU) were derived from five to six individual lungs per point.

Using semiquantitative polymerase chain reaction gene expression analysis, we defined the temporal appearance of a panel of CXCL and CCL chemokines during synchronized innate and adaptive antigen bead-elicited granuloma formation (Chiu et al., 2003). Lungs of mice were harvested and analyzed at intervals after granuloma induction. As shown in Figure 4, both innate and adaptive responses displayed the greatest expression of chemokines in the first 2–3 days of granuloma formation, corresponding to the period of robust cellular recruitment. Despite quite different antigens, the patterns and degree of chemokines produced during innate granuloma formation in response to BSA, SEA, and PPD beads were very similar, except the PPD-elicited response showed higher expression of neutrophil chemotactins, CXCL2, CXCL5, and CCL3, which correlated with greater neutrophil recruitment. This finding suggested the presence of additional Ag-related innate recognition and was consistent with the ability of mycobacterial PPD components to stimulate Toll-like receptors, TLR2 and TLR4 (Means et al., 2001).

During adaptive granuloma formation, polarization of the mycobacterial and helminth antigen bead-elicited chemokine profiles became clearly apparent. During PPD-bead elicited granuloma formation the CXCL chemokines dominated with levels increasing up to 10-fold over the innate response. In contrast, during the SEA response these remained at innate levels. Among the CCL chemokines, there was amplification of most but with a lesser degree of polarization. However, the SEA-bead elicited response displayed augmented CCL7 expression whereas the PPD response remained at innate levels. CCL7 is a ligand for the CCR3 receptor expressed by eosinophils which are a major component of the adaptive granulomatous response to SEA.

## Cytokine Regulation of Chemokine Expression during Adaptive Granuloma Formation

Chemokine expression analyses of Ag-bead-elicited granuloma formation demonstrated the presence of innate chemokine responses which were modified by superimposition of adaptive immunity. Moreover, the character of the adaptive chemokine profile differed depending on whether the adaptive cellular response was either Th1- or Th2-mediated. To directly test if Th1 and Th2-related cytokines were responsible for shaping the chemokine patterns observed during the adaptive response, mice with either type-1 PPD- or type-2 SEA-bead elicited adaptive granulomas were treated with cytokine neutralizing antibodies to IFNγ, IL-12, IL-4, IL-10, or IL-13, then chemokine transcripts were measured on day 2, during the period of most active chemokine induction. As summarized in Table 1, IFNγ depletion decreased a number of the transcripts during mycobacterial PPD-elicited type-1 granuloma formation, especially the CXCL chemokines. This was associated with a significant reduction in granuloma size measured on day 4. In the helminth SEA-elicited type-2 response, the IFNγ-dependent CXCL9–11 chemokines were also reduced but CCL4, CCL7, CCL17, and CCL22 were increased with about a 30% augmentation of granuloma cross-sectional area. This demonstrated a cross-regulatory effect of endogenous IFNγ in the type-2 response. Anti-IL-12 also reduced CXCL9-11 during the type-1 response but with a lesser reduction in granuloma size. Since IL-12 is an innate stage cytokine known to enhance IFNγ, this likely represented an effect on the IL-12 dependent component of IFNγ production, though a direct effect could not be ruled out. In the type-2 response, IL-12 depletion had no effect on CXC but increased a number of the CC chemokines without affecting overall inflammation, suggesting that endogenous IL-12 has a regulatory effect but the enhanced CC chemokine transcript expression could not further augment inflammation. Since IFNγ depletion did reduce CXC chemokines and augmented type-2 inflammation, it suggests a role for CXC chemokines in recruitment or stimulation of a population that tempers type-2 inflammation. In the type-2 response, depletion of the Th2 cytokines, IL-4, or IL-3, caused reductions of most CC with no effect on the CXC chemokine transcripts, which was associated with significant reductions in granuloma sizes. Anti-IL-13 also reduced some CC chemokines in the type-1 PPD response with augmentation of CXCL9-11, indicating a regulatory effect of underlying IL-13, but no net effect on inflammation was observed. Depletion of endogenous IL-10 which may derive from both innate and adaptive immune cells appeared to influence only a limited number of CC chemokines with no effect on overall inflammation.

**Table 1:** Effect of cytokine depletion of chemokine transcript expression and granuloma formation*.

Granuloma type and treatment	CXCL2	CXCL5	CXCL9	CXCL10	CXCL11	CCL1	CCL2	CCL3	CCL4	CCL7	CCL8	CCL11	CCL17	CCL22	XCL1	Effect on lesion area
**TYPE-1 (PPD)**																
Anti-IFN																−41%
Anti-IL-12																−14%
Anti-IL-4	ND	ND	ND	ND	ND	ND	ND	ND	ND	ND	ND	ND	ND	ND	ND	ND
Anti-IL-13																
Anti-IL-10																
**TYPE-2 (SEA)**																
Anti-IFN																+28%
Anti-IL-12																
Anti-IL-4																−38%
Anti-IL-13																−30%
Anti-IL-10																

*Cells shaded red indicates significant decreases; green indicates significant increases and white indicates no change. ND, not determined.

It should be noted that in addition to our finding of chemokine modulation by Th1- and Th2-related cytokines, recent studies of neutrophil-rich granulomas elicited by *S. japonicum* eggs show participation of Th17 cells and IL-17 dependence of neutrophil chemotactins, CXCL1, and CXCL2 (Zhang et al., 2012).

To summarize, cytokines appear to shape the spectrum and degree of chemokines produced during adaptive granuloma formation through positive and negative signaling. Polarized cytokine responses lead to different chemokine profiles presumably designed to generate a pathogen specific granulomatous response.

## Chemokine Contribution to Granuloma Formation

Published literature demonstrates consistent circumstantial expression of chemokines in animals models of granulomatous conditions and human granulomatous diseases. However, revealing their functional significance is more challenging. Because, of their potential promiscuity in receptor-binding, experiments designed to neutralize or knockout individual chemokines leave open the possibility of compensatory or redundant chemokines. The alternative approach is to focus on the relative requirement of chemokine receptors, which are less numerous than chemokines and often show restricted cellular expression. An important aspect of chemokine receptors is their dynamic nature. A particular cell may express different receptors at different maturational stages as well as alter levels of expression following activation or receptor ligation. Most studies of chemokine receptors employ chemokine receptor knockout mouse strains or specific receptor antagonists. We and others have used these approaches to assess their role in granuloma formation. Those findings are discussed below.

### CCR1

CCR1 (CD191) is a highly promiscuous receptor with reported binding to multiple CC ligands (CCL3, CCL5, CCL7, CCL8, CCL14, CCL15, CCL16, and CCL23). It is expressed by a number of cell types including macrophages and lymphocyte populations. When type-1 PPD- or type-2 SEA-bead elicited adaptive granulomas were elicited in CCR1 knockout (CCR1^−/−^) mice, there was little effect on overall sizes of secondary lesions. However, there were subtle but distinct effects on the type-1 response. The latter unlike the type-2 lesion is characterized by a component of natural killer (NK) cells, which was absent in CCR1^−/−^ mice. In addition, there was reduced production of Th1-associated cytokines by draining lymph node cultures (Shang et al., 2000). In a separate study, Gao et al. (2000) reported impairment neutrophil recruitment and abrogation of the granulomatous response to primary injection of whole *S. mansoni* eggs in CCR1^−/−^ mice. When we tested the innate granulomatous response to PPD- and SEA-beads, we again showed impaired NK cell recruitment as well as compromised neutrophil recruitment (Chiu et al., 2004). Taken together, the findings suggest a role for CCR1 in the innate response, which may partially influence the quality of subsequent adaptive responses.

### CCR2

Like CCR1, CCR2 (CD192) is a promiscuous receptor, binding CCL2, CCL7, CCL8, CCL13, and CCL16. A wide variety of cell types are reported to express CCR2 including monocytes, lymphocytes, and non-leukocytic cells. When type-1 PPD- or type-2 SEA-bead elicited adaptive granulomas were elicited in CCR2 knockout (CCR2^−/−^) mice, there was impaired monocyte/macrophage recruitment especially during the initial stages of granuloma formation. In the type-1 response this was associated with notable reductions in Th1 cytokine production by draining lymph node cultures and while there was partial reduction of IL-4 producing capacity in the type-2 response the Th2 cytokine profile remained largely intact (Boring et al., 1997; Warmington et al., 1999). An analysis of innate granuloma formation in CCR2^−/−^ mice similarly showed impaired monocyte/macrophage recruitment in both PPD- and SEA-bead elicited lesions. In addition, both showed significant reductions in granuloma associated myeloid dendritic cells (mDCs), thus providing a potential link between recruitment and adaptive phase cytokine defects (Chiu et al., 2004). A similar monocyte/macrophage recruitment defect has been reported in a model of zymosan-elicited granuloma formation (Jinnouchi et al., 2003). Together the findings suggest a critical role for CCR2 in the mobilization of the innate monocyte/macrophage and mDC component of granulomas. The lack of which can significantly influence subsequent adaptive stage events, mainly the Th1 response.

### CCR3

CCR3 (CD193) is another promiscuous receptor binding CCL3, CCL5, CCL7, CCL11, CCL13, CCL14, CCL15, CCL24, and CCL26. It is highly expressed by eosinophils and basophils as well as lymphocyte subpopulations and some non-leukocytic cells. No systematic studies of granuloma formation in CCR3 knockout mice have been reported. However, we have tested the role of two its ligands, CCL7 and CCL11 (eotaxin). Transcript analyses in those studies showed induction of CCR3, CCL7, and CCL11 during both type-1 PPD- and type-2 SEA-bead elicited adaptive granuloma formation, but expression of all was greater in the latter and augmented expression was dependent on IL-4. *In vivo* CCL7 and CCL11 depletions revealed that CCL7 and not CCL11 contributed to eosinophil recruitment in the type-2 response (Ruth et al., 1998; Shang et al., 2002). These data provided indirect evidence of differential participation of CCR3 agonists and demonstrated a role for selected chemokines in shaping granuloma cellular composition.

### CCR4

CCR4 (CD194) has two known agonist ligands CCL17 and CCL22. The receptor is reportedly expressed by a variety of leukocytic and non-leukocytic populations. Among T cells it has been mainly associated with skin homing CD4+ effector memory, Th2 effector, and T regulatory cells. Constitutive expression of CCL17 and CCL22 is detectable among myeloid DCs and is upregulated by inflammatory stimuli (Penna et al., 2002). As described above, these ligands are induced during innate and adaptive type-1 PPD- and type-2 SEA-bead elicited granuloma formation. When adaptive granulomas were elicited in CCR4 knockout (CCR4^−/−^) mice, there was partial abrogation of the type-1 response and decreased local IFNγ production. The inflammation associated with type-2 granulomas was largely unaffected but showed reductions of local IL-5 and IL-13 production (Freeman et al., 2006). These studies indicated that in the mouse CCR4 participation was not restricted to Th2 dominant responses. Additional *in vivo* and *in vitro* experiments suggested that CCR4 was required for effector memory cell activation by mDCs. Thus, CCR4 may allow for optimal contacts between effector cells and mDCs under homeostatic and challenge conditions.

### CCR5

CCR5 (CD195) is a promiscuous receptor-binding CCL4, CCL5, CCL8, CCL11, CCL14, and CCL16 as agonists. It has been extensively studied due to its ability to act a coreceptor for human immunodeficiency virus (HIV). CCR5 is expressed on T cells, macrophages, mDCs, and some non-leukocytic cells. Among T cells it reportedly shows biased expression by CD4+ Th1 and CD8+ T cells, however most reports suggest CCR5 is dispensable for mounting resistance to variety of pathogen challenges. In regard to granuloma formation, we observed no effect of CCR5 knockout on the innate response to either PPD- or SEA-beads (Chiu et al., 2004). However, Souza et al. (2011) demonstrated significant exacerbation of the Th2-mediated granulomatous response to *S. mansoni* eggs. This observation would be consistent with our finding that depletion of CCL5, a potent agonist of CCR5, augments type-2 granuloma formation (Chensue et al., 1999). Taken together, these studies suggest the presence of CCR5-dependent immunoregulation of type-2 adaptive granuloma formation.

### CCR6

Unlike other CC chemokine receptors, the gene for CCR6 (CD196) is carried on a different chromosome (human chromosome 6, mouse chromosome 17) than the others which are clustered (human chromosome 3, mouse chromosome 9). It is also unique in that it has only one known chemokine agonist ligand, CCL20. CCR6 is expressed by mDCs, mature B cells, subpopulations of memory/effector CD4+ and CD8+ T cells, but not plasmacytoid DCs or naïve T cells. Among CD4+ T cells, CCR6 has been associated with Th1 and Th17 subpopulations. While the ligand for CCR6 has been reported in models of granuloma formation (Qiu et al., 2001), there are as yet no systematic functional analyses. Despite its expression by immature mDCs, no defect in local mDC recruitment was observed during the innate granulomatous response to PPD- or SEA-beads elicited in CCR6 knockout (CCR6^−/−^) mice (Chiu et al., 2004). In preliminary studies of adaptive type-1 PPD- and type-2 SEA-bead elicited granuloma formation, CCR6^−/−^ mice displayed no defects in type-1 inflammation and only a partial impairment of eosinophil recruitment in the type-2 response. However, alterations in draining lymph node cytokine and immunoglobulin induction events were noted (unpublished observations). A number of studies point to a role for CCR6 in mucosal and lymphoid tissue events (Ito et al., 2011), hence there may be less contribution to interstitial granulomatous responses. However, CCR6-mediated effects on the adaptive immunity could influence some aspects of granuloma formation.

### CCR8

CCR8 (CDw198) has a limited agonist complement, CCL1, CCL8 (mouse), CCL16, and is expressed by T cells and possibly macrophage subsets. Among T cells it has been associated with Th2 and T regulatory populations. There are limited studies examining the contribution of CCR8 to granuloma formation. We performed detailed analyses of type-1 PPD- and type-2 SEA-bead elicited adaptive granuloma formation in CCR8 knockout (CCR8^−/−^) mice (Chensue et al., 2001; Freeman et al., 2005). Those studies showed the CCR8 was required for establishment of a normal eosinophil-rich type-2 granuloma, but was not required for the type-1 granulomatous response. The critical CCR8+ population was identified as a CD4+ IL-10+ regulatory T cell that appeared to enhance expression of the Th2 response likely by tempering cross-regulatory signals.

### CXCR4

CXCR4 (CD184) has one known chemokine agonist, CXCL12, and is expressed by a variety of leukocytes including T cells as well as non-leukocytic cells. In addition to T cell chemotaxis, CXCR4 appears to play roles in regulating stem cell homing to the bone marrow, HIV entry into cells and tumor metastasis. Since CXCR4 knockout is lethal, we used a specific CXCR4 receptor antagonist, AMD3465, to test its effects on type-1 PPD- and type-2 SEA-bead elicited granuloma formation (Hu et al., 2006). CXCL12 transcripts are induced locally and in draining lymph nodes upon granuloma elicitation with the greatest expression in the adaptive type-2 response. Accordingly, treatment had no effect on the innate or the adaptive type-1 responses, but the type-2 adaptive response was significantly abrogated with reductions of both eosinophils and mononuclear phagocytes. In addition, Th2 cytokine production by draining lymph node cultures was notably compromised. Thus, CXCR4 showed biased participation in establishing the type-2 adaptive granulomatous response. Precise mechanisms remain to be determined.

## Conclusion

Using models of synchronized innate and adaptive pulmonary granuloma formation we have demonstrated a role for chemokines in the development of these lesions. The emerging picture is one in which different chemokines contribute to different stages, components, and functional aspects of granulomas. Conceptualizing and classifying granulomas in terms of innate and adaptive responses allows for more precise interpretation of cellular events. As described above, some chemokines contribute more to the innate component of the response whereas others are invoked by adaptive stage molecular signals amplifying and altering granuloma cellular composition to provide functions needed to contain and or eliminate an insulting agent. As major mediators of the inflammation, chemokines are a potential target for therapeutic manipulations to temper or optimize the granulomatous response.

## Conflict of Interest Statement

The authors declare that the research was conducted in the absence of any commercial or financial relationships that could be construed as a potential conflict of interest.
